# The promise of GLP-1 receptor agonists for neurodegenerative diseases

**DOI:** 10.1172/JCI194745

**Published:** 2026-02-16

**Authors:** Dilan Athauda, Nigel H. Greig, Wassilios G. Meissner, Thomas Foltynie, Sonia Gandhi

**Affiliations:** 1 The Francis Crick Institute, London, United Kingdom.; 2 Department of Clinical and Movement Neurosciences, UCL Queen Square Institute of Neurology, London, United Kingdom.; 3 Drug Design and Development Section, Translational Gerontology Branch, Intramural Research Program, National Institute on Aging, NIH, Baltimore, Maryland, USA.; 4 CHU Bordeaux, Service de Neurologie des Maladies Neurodégénératives, IMNc, Bordeaux, France.; 5 University de Bordeaux, CNRS, IMN, UMR 5293, Bordeaux, France.; 6 Department of Medicine, University of Otago, Christchurch, and New Zealand Brain Research Institute, Christchurch, New Zealand.

## Abstract

Glucagon-like peptide-1 receptor agonists (GLP-1RAs), established therapies for type 2 diabetes and obesity, are increasingly recognized for their potential in neurodegenerative diseases. Preclinical studies across diverse neurodegenerative conditions consistently demonstrate neuroprotective effects of GLP-1RAs, including reduced protein aggregation, enhanced autophagy, improved mitochondrial function, suppression of neuroinflammation, and preservation of synaptic integrity. Epidemiological analyses further suggest reduced incidence of dementia, Parkinson disease, and multiple sclerosis among long-term GLP-1RA users. Early human trials provide signals of target engagement, such as preserved cerebral glucose metabolism, altered inflammatory biomarkers, and slowed brain atrophy, although clinical outcomes to date remain mixed and trials in rarer disorders are sparse. Translation is constrained by uncertainty around optimal molecule choice, CNS penetrance, tolerability, adherence, and heterogeneity of response. Furthermore, next-generation dual and triple agonists may offer enhanced efficacy but remain untested in neurodegeneration. Conceptually, GLP-1RAs share pleiotropic effects with exercise — one of the few interventions with proven disease-modifying potential — by enhancing insulin signaling, stabilizing mitochondria, reducing inflammation, and promoting synaptic plasticity. This overlap highlights their promise as “pharmacological analogues of exercise,” and underscores the need for biomarker-driven, disease-specific trials to establish whether GLP-1RAs can deliver durable disease modification across the spectrum of neurodegenerative diseases.

## Introduction

Neurodegenerative diseases (NDDs) such as Alzheimer disease (AD), Parkinson disease (PD), dementia with Lewy bodies (DLB), multiple system atrophy (MSA), Huntington disease (HD), amyotrophic lateral sclerosis (ALS), and multiple sclerosis (MS) are collectively characterized by progressive neuronal loss leading to impairments in cognition, motor control, and behavior. Their prevalence is rising sharply and is projected to surpass cancer as the second leading cause of death by 2040 (1). Although each condition has distinct pathological features, they converge on shared, interlinked biological “hallmarks”: pathological protein aggregation, synaptic and network dysfunction, impaired proteostasis, cytoskeletal abnormalities, mitochondrial dysfunction, inflammation, and ultimately neuronal death.

A substantial body of evidence now demonstrates that these hallmarks are strongly modulated by systemic metabolic health. Type 2 diabetes mellitus (T2DM), defined by insulin resistance (IR), hyperglycemia, and altered lipid metabolism, increases the risk of several NDDs (2). Epidemiological studies show that people with T2DM have approximately double the risk of AD (3); an approximately 40% increased risk of developing PD (4–6); and are approximately 1.5 times more likely to develop MS (7), with longer duration and poorer glycemic control conferring greater risk. Conversely, T2DM appears to reduce ALS risk in older adults (8–10), though this may reflect effects of insulinotropic medications rather than diabetes itself (11). Importantly, comorbid T2DM or elevated peripheral IR accelerate disease progression in AD and PD independently of vascular disease, with higher peripheral IR predicting conversion from mild cognitive impairment to AD (12, 13) and faster motor and cognitive decline in PD (14, 15), thereby implicating IR as a modifier of NDD biology rather than a simple comorbidity.

A convergent mechanism linking these conditions is brain insulin resistance (BIR). In the CNS, insulin regulates synaptic plasticity, mitochondrial function, neuronal survival, and glial activation. In BIR, inhibitory serine phosphorylation of IRS-1 (S312/S616/S636) disrupts PI3K/Akt signaling, deactivating downstream insulin signaling. This leads to impaired synaptic plasticity and long-term potentiation (LTP) (16, 17), tau hyperphosphorylation (18–21), reduced clearance of Aβ (22–24) and α-synuclein (25, 26), and microglial activation (27, 28), and impaired mitochondrial function (29, 30). These processes form a self-reinforcing loop; IR-driven mitochondrial stress and impaired mitophagy exacerbate Aβ (31, 32), tau (18), and α-synuclein pathology (25), which in turn further impairs insulin signaling (33). BIR has been demonstrated in postmortem studies of patients with PD (34), AD (35), DLB (36), and MSA (37), associated with dopaminergic depletion in the caudate and ventral striatum (14, 38), frontotemporal cortical atrophy (39, 40), and cerebral glucose hypometabolism (41). Similar metabolic defects occur in HD (42, 43) and MS (44).

These insights have intensified interest in therapies that restore central metabolic resilience. Among these, glucagon-like peptide-1 receptor agonists (GLP-1RAs) — developed to treat T2DM and obesity — are uniquely positioned as potential disease-modifying agents. GLP-1 is secreted by intestinal L cells and by preproglucagon neurons in the nucleus tractus solitarius, which project widely to areas governing cognition, motor control, reward, autonomic regulation, and energy homeostasis (45, 46). GLP-1Rs are expressed in the hypothalamus, hippocampus, cortex, and brainstem, as well as limbic and reward-related circuits (47). Activation of GLP-1R engages cAMP/PKA and PI3K/Akt pathways overlapping with insulin receptor signaling. Through these mechanisms, GLP-1RAs activate multiple pathways, reduce inflammation, improve mitochondrial function, modulate proteostasis, and promote synaptic plasticity (48–52), a profile well suited to the multifactorial nature of NDDs.

Across diverse preclinical models and early human trials, GLP-1RAs demonstrate broad neuroprotective effects and signals of clinical benefit, with next-generation agents aiming to further enhance central penetrance and potency. Nevertheless, important uncertainties remain, including the degree of brain penetrance across individual agents, the relative contributions of central versus peripheral mechanisms, biomarkers of target engagement, and the patient subgroups most likely to benefit.

In this Review, we evaluate the translational evidence supporting GLP-1RAs as candidate disease-modifying therapies for NDDs, describe mechanistic pathways linking metabolic dysfunction to neuronal vulnerability, and outline remaining challenges and research priorities in this rapidly evolving field.

## Central mechanisms of GLP-1RAs in neurodegeneration

### Regulation of protein aggregation and proteostasis.

Pathological protein aggregation is a unifying hallmark of NDDs: Aβ and tau in AD, α-synuclein in PD and MSA, mutant huntingtin in HD, and TDP-43 in ALS. These assemblies impair multiple cellular processes including synaptic, mitochondrial, and autophagy–lysosomal function, and activate inflammation, making restoration of proteostasis a central therapeutic goal.

**Figure 1:**
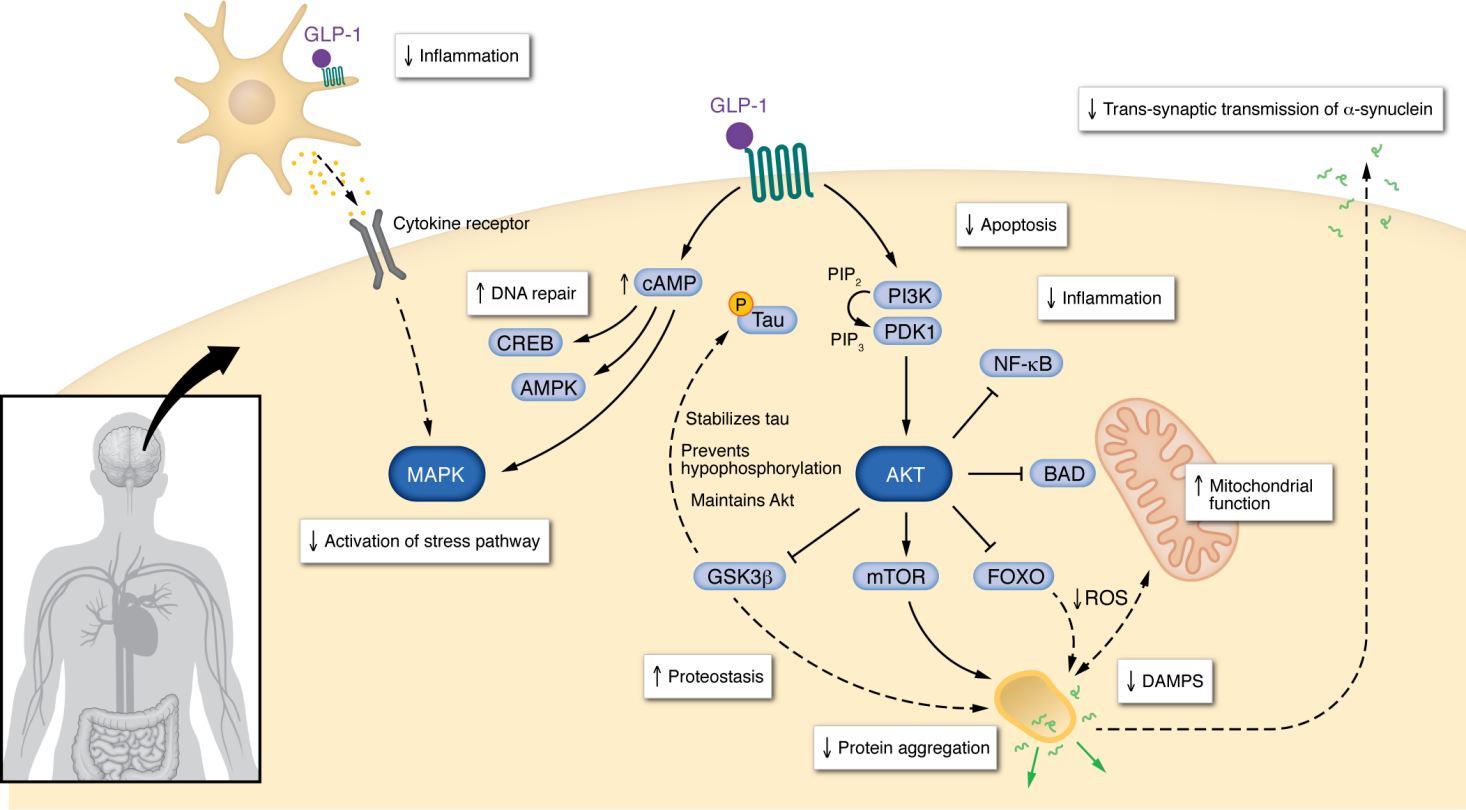
Central mechanisms of GLP-1RAs. GLP-1RAs engage PI3K/AKT and cAMP/PKA pathways to modulate autophagy and improve mitochondrial function and redox homeostasis. Collectively, these effects promote protein clearance and reduce cellular stress that drives aggregation. GLP-1RAs also exert direct antiinflammatory effects on glia. These pleiotropic actions converge to preserve neuronal viability and network function.

GLP-1R activation engages PI3K/Akt and cAMP/PKA pathways that converge on kinases that modulate autophagy, including mTOR, AMPK, and transcription factor EB (TFEB). GLP-1RAs also upregulate sirtuin-1 (SIRT1) indirectly (53), which deacetylates autophagy-related proteins and promotes autophagosome formation. Complementary improvements in mitochondrial function and redox homeostasis further support proteostasis. Through these mechanisms, GLP-1RAs promote protein clearance and reduce cellular stress that drives aggregation (Figure 1).

Preclinical evidence shows broad anti-aggregation effects of GLP-1RAs. GLP-1RAs reduce tau phosphorylation and Aβ plaque burden in AD models (54–56), attenuate α-synuclein pathology in PD models (57) and in induced pluripotent stem cell–derived (iPSC-derived) dopaminergic neurons with PD-linked *SNCA* mutations (26), and lower mutant huntingtin aggregates in a mouse model of HD (58). Data in ALS models are less robust but suggest GLP-1RAs induce partial mitigation of TDP-43 pathology (59).

Clinical evidence supporting these effects remains limited. In a phase II trial of 60 individuals with PD, 48 weeks of exenatide, a first-generation GLP-1RA, was associated with reduced oligomeric α-synuclein in the cerebrospinal fluid (CSF) compared with placebo, alongside motor benefits (26, 60). However, these results were not replicated in a larger multicenter trial conducted over 96 weeks (61). In a study of 18 people with AD, exenatide treatment for 18 months was associated with lower plasma-sourced, neuron-derived extracellular vesicle (NDEV) Aβ42 levels, although all other fluid biomarkers remained unchanged (62). Consistent effects on tau- or p-tau–related biomarkers remain unproven.

Overall, these results indicate GLP-1RAs can influence a number of proteinopathies across disease models, although more understanding is required to determine when in the course of disease proteostasis can be modulated to bring about reduced neuronal loss and clinical benefit. Translation to clinical benefit will require biomarker-driven studies incorporating fluid and imaging measures of proteostasis.

### Restoring energy metabolism and mitochondrial function.

Defective energy metabolism is a consistent feature of NDDs, encompassing mitochondrial dysfunction, altered substrate utilization, and impaired hypothalamic regulation. Familial PD genes (Parkin [*PRKN*, also known as *PARK2*] and *PINK1*) implicate impaired mitophagy in disease progression (63), fluorodeoxyglucose PET (FDG-PET) studies show consistent cerebral glucose hypometabolism (64), and hypothalamic dysfunction has been linked to ALS and HD (65). These abnormalities support the concept that energy homeostasis failure is an early and upstream driver of neurodegeneration.

GLP-1RAs can target these abnormalities at multiple levels. Via AMPK/SIRT1/PGC-1α signaling, they promote mitochondrial biogenesis (66–68) and enhance PINK1- and Parkin-mediated mitophagy. These effects, together with upregulation of antioxidant modulators such as Nrf2, reduce ROS generation (69–71). In glia, AMPK/SIRT1/PGC-1α signaling counters proinflammatory metabolic shifts, e.g., glycolytic reliance in astrocytes, improving neuronal support and functional outcomes in transgenic AD mice (72). Furthermore, GLP-1RAs can affect hypothalamic regulation; for example, liraglutide and exendin-4 normalized glucose metabolism and prevented weight loss in a transgenic HD model (58).

Clinical data are limited but support these effects. In patients with AD, 6 months of liraglutide increased blood-brain glucose transfer (likely via GLUT upregulation) (73), and stabilized cerebral glucose metabolism on FDG-PET (74). Whether these metabolic effects translate into durable clinical benefit or arise from direct CNS actions versus systemic metabolic improvement remains uncertain.

### Mitigating DNA damage and RNA dysregulation.

Neurons are highly vulnerable to DNA damage caused by ROS from mitochondrial metabolism, while upstream defects in RNA processing involving defects in splicing, transport, and stress granule dynamics can disrupt protein homeostasis and promote aggregation. These nucleic acid defects are central features of multiple NDDs.

GLP-1RAs have recently been shown to mitigate these processes by enhancing DNA repair and stabilizing RNA metabolism. GLP-1RAs can enhance neuronal DNA repair capacity through the base excision repair pathway (75). In an ischemic stroke model, exendin-4 enhanced the activation of PI3K/Akt/CREB signaling, leading to upregulation of APE1, a key enzyme responsible for resolving oxidized bases and single-strand breaks, which reduced nuclear DNA fragmentation and preserved neuronal viability (75, 76). In addition, by improving mitochondrial function through AMPK/SIRT1 signaling, GLP-1RAs reduce ROS generation, indirectly lowering DNA and RNA damage burden.

Overall, GLP-1RAs clearly enhance DNA repair and reduce oxidative stress, but their impact on RNA-processing defects remains uncertain. Establishing their impact on nucleic acid pathology will require studies in proteinopathies such as ALS, FTD, and HD.

### Regulating glial activation and neuroinflammation.

Neuroinflammation is a key hallmark of NDDs, characterized by chronic activation of microglia and astrocytes, excessive cytokine release, and infiltration of peripheral immune cells. Although inflammatory responses are initially protective, sustained glial activation promotes oxidative stress and synaptic injury, and accelerates protein aggregation, which itself exacerbates inflammation, perpetuating a vicious cycle.

GLP-1Rs are expressed on glial cells (77, 78), which allows GLP-1RAs to exert direct antiinflammatory effects. Activation of GLP-1R enhances Akt-dominant (neuroprotective) signaling and avoids prolonged MAPK/JNK signaling that drives cytokine release. GLP-1R–mediated activation of the cAMP/PKA and PI3K/Akt pathways inhibits NF-κB, and thus reduces expression of proinflammatory cytokines such as TNF-α, IL-1β, and IL-6 (79). GLP-RAs also activate AMPK, and through downstream SIRT1 also inhibit NF-κB translocation and thus indirectly suppress proinflammatory gene transcription (80), leading to promotion of autophagy and mitochondrial stability, thereby reducing damage-associated molecular patterns that sustain microglial activation (81). In addition, GLP-1RAs inhibit the NLRP3 inflammasome (which is implicated in multiple NDDs; ref. 82), diminishing activation of its downstream cytokine cascade and reducing pyroptosis (83). Another emerging mechanism involves the cGAS/STING pathway; mitochondrial stress and defective autophagy cause cytosolic leakage of mtDNA, activating STING and proinflammatory cytokines, and STING dysfunction has been identified as a key player in NDDs (84, 85). By enhancing autophagy and stabilizing mitochondria indirectly via AMPK and SIRT, GLP-1RAs may also act as indirect suppressors of pathological STING activation.

Preclinical models support these mechanisms. GLP-1RAs reduce microglial activation and cytokine release in PD (86), AD, and MS models (87), with parallel improvements in behavior. Clinical evidence, although still emerging, aligns with these findings. In people with T2DM, exenatide treatment reduced serum levels of inflammatory proteins linked to AD risk (88), and studies with liraglutide or sitagliptin (a DPP-IV inhibitor that elevates GLP-1 levels) reported lower cytokine levels and modest cognitive improvements (89). However, direct biomarker evidence in NDD populations is limited, and the relative contributions of central versus systemic immune modulation remain uncertain.

Collectively, GLP-1RAs exert immunomodulatory effects through multiple mechanisms, including reducing NF-κB and inflammasome signaling, activating SIRT1/AMPK networks, and attenuating inflammatory glial phenotypes. Key uncertainties include how best to measure these effects in vivo and the disease stages at which inflammatory modulation is most beneficial.

### Restoring synaptic function and network integrity.

Synaptic dysfunction is one of the earliest features of NDDs, preceding neuronal loss and driving network dysfunction. Normal synaptic activity can be disrupted by mitochondrial dysfunction, aberrant glutamate signaling, and toxic protein aggregates, resulting in synaptic hyperexcitability, loss of dendritic spines, and impaired plasticity.

GLP-1RAs act on several of these mechanisms. Activation of cAMP/PKA/CREB signaling upregulates brain-derived neurotrophic factor (BDNF) and stabilizes dendritic spines (90). In hippocampal preparations, exendin-4 and liraglutide restore LTP suppressed by Aβ and tau (56, 91), and GLP-1RAs also normalize calcium handling and limit glutamate excitotoxicity (79, 92, 93). By enhancing autophagy and proteostasis, they facilitate clearance of toxic protein oligomers at pre- and postsynaptic sites, thereby preserving function (94, 95). Together, these effects are associated with preservation of synaptic proteins such as synaptophysin and PSD-95 in AD and PD models (96).

In human studies, liraglutide treatment in AD enhanced cerebral glucose metabolism and showed signals of preserved network activity (97). However, direct evidence for true synaptic rescue remains limited.

Overall, GLP-1RAs may affect multiple pathways that influence synaptic integrity, and these effects, though not yet fully validated in humans, provide a basis for their potential to slow network dysfunction across NDDs.

### Restoring gut-immune-brain homeostasis.

**Figure 2:**
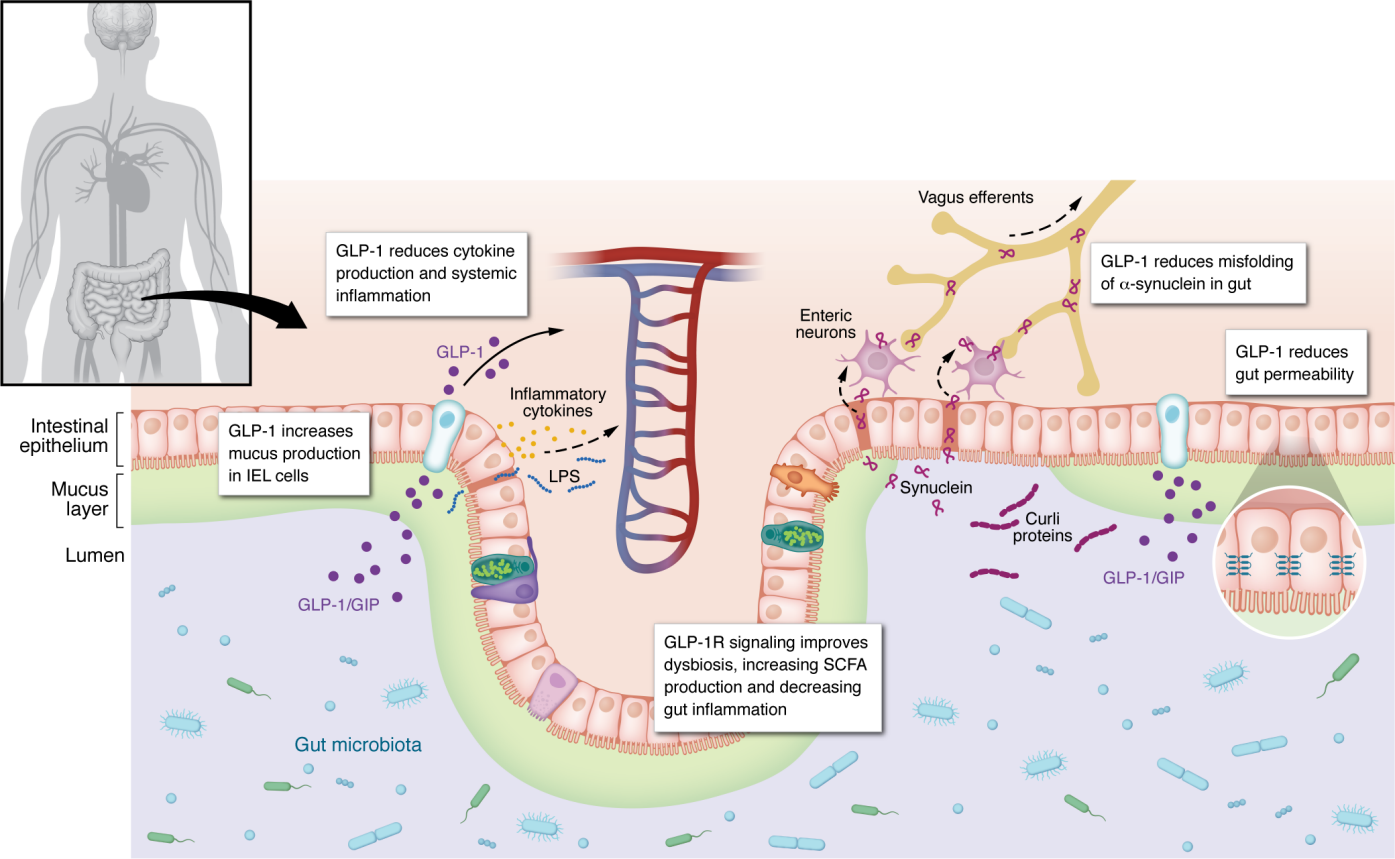
Modulation of the gut-immune-brain axis by GLP-1RAs. GLP-1Rs are expressed throughout the gut-immune-brain network, enabling GLP-1RAs to restore homeostasis at multiple levels. GLP-1RAs reshape microbiota composition toward beneficial taxa. GLP-1RAs also improve gut and BBB integrity, preventing leakage of microbial metabolites into the CNS and reducing inflammation. Improved barrier function may also prevent bacterial amyloids, such as *E*. *coli* curli proteins, from interacting with human amyloids and accelerating their aggregation. Together, these actions support homeostasis across the gut-brain axis. IEL, intraepithelial lymphocyte.

The gut microbiota–immune-brain axis is a complex bidirectional network connecting microbial communities, the vagus nerve, the neuroendocrine system, and immune pathways (Figure 2). Dysbiosis, characterized by a reduction in microbiota diversity, leads to loss of “beneficial” bacteria that produce antiinflammatory short-chain fatty acids (SCFAs) and has been observed across AD, PD, and MS (98–101). This imbalance promotes increased intestinal and blood-brain barrier (BBB) permeability, allowing inflammatory molecules from the intestine to reach the brain and vice versa, and can cause systemic and neuroinflammation; and facilitating entry of microbial metabolites into the CNS. For example, bacterial amyloids, such as *E*. *coli* curli proteins, can directly interact with human amyloids, and potentially accelerate aggregation of human Aβ and α-synuclein in AD and PD (102, 103). In addition, the gut-derived metabolite imidazole propionate has been shown to drive α-synuclein pathology in preclinical and patient-derived PD models, providing a direct link between dysbiosis and α-synuclein aggregation (103). Dysbiosis in PD toxin models is also accompanied by reduced GLP-1 levels in colon and brain, reversible with probiotics, further linking this axis to disease vulnerability (104).

**Figure 3:**
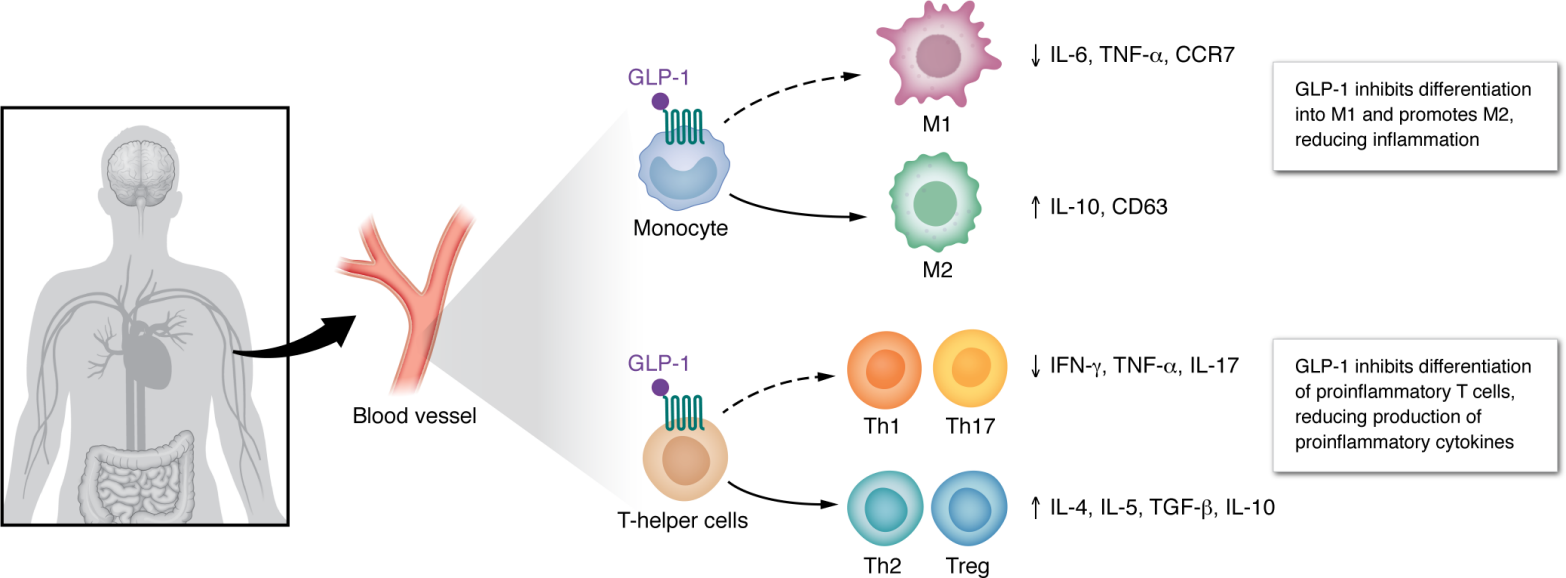
Systemic antiinflammatory effects of GLP-1RAs. GLP-1RAs can act directly on GLP-1Rs expressed on immune cells. In macrophages, this inhibits polarization toward an M1-like state, and promotes production of IL-10. In T cells, GLP-1R activation decreases differentiation into Th1 and Th17 cells, and promotes differentiation toward Th2 and Treg identities.

GLP-1Rs are expressed throughout the gut-immune-brain network, enabling GLP-1RAs to restore homeostasis at multiple levels. At the gut barrier, GLP-1RAs improve epithelial integrity, reduce tight junction permeability, and suppress endotoxin translocation, thereby lowering systemic LPS-driven inflammation (105). GLP-1RAs also shift microbiota composition, increasing beneficial SCFA-producing taxa such as *Akkermansia* and *Bifidobacterium*, which may alleviate systemic inflammation (106–108). Within the immune system, they inhibit NF-κB and NLRP3 inflammasome activation, promote antiinflammatory IL-10 release, and promote macrophages and microglia toward antiinflammatory phenotypes (109, 110) (Figure 3), which could indirectly reduce cross-seeding of Aβ and α-synuclein initiated in the gut.

Together, these pleiotropic effects highlight the gut-immune-brain axis as both a site of vulnerability and a therapeutic target in NDDs. However, clinical data remain scarce, and it is unclear whether microbiome changes reflect direct GLP-1R signaling or secondary metabolic effects. Defining these relationships will be essential for translational progress.

## GLP-1 therapies in neurodegenerative diseases

As described above, GLP-1RAs engage multiple convergent pathways implicated across NDDs, from proteostasis and mitochondrial quality control to neuroinflammation, synapse function, and gut-brain-immune interactions. The next step is to consider how these pleiotropic actions translate within specific disease contexts (Table 1).

### AD.

GLP-1RAs consistently demonstrate neuroprotective effects in AD models. Exenatide, lixisenatide, liraglutide, dulaglutide, and semaglutide have been shown to improve learning and memory while reducing Aβ/tau pathology, neuroinflammation, and synaptic/metabolic deficits in rodent models of sporadic toxin-induced neuronal injury (Aβ, streptozotocin, okadaic acid) (54, 111–120), and in multiple transgenic strains (5×FAD, 3×Tg, APP/PS1, Tg2576, APP/PS1/Tau) (121, 121–133). Human AD brain organoids treated with semaglutide similarly showed reduced Aβ, phosphorylated tau, and GFAP expression (134). These effects are attributed to restoration of neuronal insulin signaling, inhibition of GSK3β, reducing NF-κB/NLRP3-induced inflammation, and normalization of mitochondrial function and mitophagy. Notably, some studies report a lack of response (135).

Emerging human data suggest that GLP-1R modulation is relevant to AD risk and pathology. *GLP1R* polymorphisms (rs10305420, rs6923761) associate with increased AD risk and lower levels of CSF Aβ42/40, and higher levels of p-tau (136). A meta-analysis of 23 clinical studies showed GLP-1RA use was associated with reduced dementia incidence (137), and large retrospective cohorts report lower AD risk in obese patients treated with GLP-1RAs (138). In addition, indirect evidence from a peripheral proteomic study of 2,000 obese individuals prescribed semaglutide showed that semaglutide downregulated tenascin-C (TNC) and upregulated progranulin (GRN), both of which may reduce AD-related pathology (139).

A number of small clinical trials directly evaluating GLP-1RAs in AD have been completed. Early small trials of liraglutide (*n =* 38) and exenatide (*n =* 18) reported no significant cognitive or biomarker effects, although exploratory analyses indicated preserved cerebral glucose metabolism (74) and reduced NDEV Aβ42 (62). More recently, a phase IIb study in AD (*n =* 206) reported 12 months of treatment with liraglutide slowed cognitive decline (ADAS-EXEC) by approximately 18% and attenuated cortical atrophy by approximately 50% (140).

In summary, preclinical evidence supports a strong class effect of GLP-1RAs in AD, but clinical outcomes remain equivocal. Despite encouraging signals (stabilized glucose metabolism, reduced atrophy, favorable proteomic shifts), definitive evidence of disease modification is lacking. Larger, biomarker-driven studies will be needed and, in this regard, two large clinical trials evaluating semaglutide in early-stage symptomatic AD reported to be negative (141).

### PD.

Preclinical studies consistently show neuroprotective effects of GLP-1RAs in PD models. In toxin-induced rodent models of PD (MPTP, 6-OHDA, rotenone, LPS), exendin-4 (142–145), liraglutide (146, 147), lixisenatide (146), and semaglutide (57, 148) reduced dopaminergic neuron loss, preserved striatal terminals, and improved motor performance, in association with stabilization of mitochondria, reduced oxidative stress, and reduced inflammation. In more disease-relevant α-synuclein models (A53T transgenic and preformed fibril mice), PEGylated exendin-4 (NLY01, a modified form of exenatide) improved survival, motor behavior, and pathology (149). In the MitoPark mouse, one of the few preclinical models of progressive PD, a clinically translatable dose of exendin-4 reduced the rate of dopaminergic neuronal loss and accompanying motor dysfunction (150), and mitigated mitochondrial dysfunction and loss (151). In human iPSC-derived dopaminergic neurons from PD patients with *SNCA* mutations, exendin-4 restored insulin signaling, improved mitochondrial function, and reduced α-synuclein aggregation via PI3K/Akt/mTOR activation (26).

Epidemiological data are consistent in demonstrating that users of GLP-1RAs have reduced risk of developing PD, ranging from 25%–50% risk reduction compared with users of other oral T2DM medications (138, 152, 153). However, clinical trial results have been mixed. Two early exenatide studies suggested motor and cognitive benefits that persisted beyond drug washout (154–156), accompanied by CSF detection of the drug and enhanced brain insulin signaling (157). In contrast, subsequent larger studies utilizing NLY01 (158) failed to show benefit. Most recently, the Exenatide-PD3 phase III trial failed to show any advantage in motor or nonsymptoms over placebo (61), and questions remain about whether a subgroup of patients with PD may be more likely to respond to this therapy. More encouragingly, a phase II randomized controlled trial (RCT) of lixisenatide (*n =* 156) reported slower motor decline over 12 months (159), while liraglutide improved nonmotor symptoms but not motor endpoints (160).

Overall, strong preclinical data and early biomarker signals provide an encouraging rationale for GLP-1RAs in PD. In addition to neurotrophic and protective actions in dopaminergic neuron–rich brain regions and attenuation of locomotor dysfunction, amelioration of L-DOPA–induced dyskinesia has been reported across PD rodent models (161, 162). However, clinical outcomes have been inconsistent. Key priorities for future trials include selecting molecules with proven CNS penetration together with enriching for biologically plausible subgroups and incorporating biomarkers to distinguish disease-modifying effects from those confounded by weight loss, tolerability, or unblinding.

### DLB.

DLB shares extensive clinical and pathological overlap with PD, with both disorders characterized by α-synuclein aggregation and dopaminergic dysfunction, leading some to suggest they represent points along a single Lewy body disease spectrum (163). Importantly, BIR markers have been observed in patients with DLB, further linking disordered insulin signaling with this synucleinopathy (36). Epidemiological data in diabetic populations indicate that people taking GLP-1RAs were about 40% less likely to develop DLB, as compared with those not taking them (138). Although there are no direct models of DLB, evidence can be extrapolated from PD models and clinical studies, which (as above) demonstrate GLP-1RAs exert broad neuroprotective actions: attenuating pathological protein aggregation, improving impaired insulin signaling, and reducing neuroinflammation. These effects directly target key DLB pathologies such as widespread microglial activation observed early in the disease and may mitigate α-synuclein–mediated neurodegeneration. Although GLP-1RAs have not yet been tested directly in DLB, mixed but encouraging findings from PD trials include potential cognitive benefits (154, 155). Due to encouraging signals from PD-related studies, and in the absence of direct DLB trial data, an international Delphi consensus has prioritized GLP-1RAs as candidates for repurposing in DLBs, underscoring the need for translational studies to confirm their therapeutic efficacy (164).

### MSA.

As a synucleinopathy with metabolic dysfunction and rapid progression, MSA represents a logical but underexplored target for GLP-1RAs. Pathological processes such as oligodendroglial insulin signaling defects, glial inflammation, mitochondrial stress, and impaired proteostasis overlap with pathways modulated by GLP-1R stimulation.

Preclinical evidence is limited. In a PLP-SYN mouse model, high-dose exendin-4 reduced markers of BIR, lowered striatal α-synuclein burden, and preserved dopaminergic neurons, but failed to halt progressive motor decline (37).

Clinical data are also sparse. A proof-of-concept, open-label trial of weekly exenatide (2 mg, 48 weeks, 50 patients) showed an advantage in the unified multiple system atrophy rating scale (UMSARS, parts I and II combined score) at 48 weeks compared with best medically treated participants, but no difference in more objective secondary measures or biomarkers (neurofilament light chain [NfL], CSF α-synuclein oligomers) (165).

In summary, although biological plausibility is strong, evidence in MSA is restricted to one animal model and one small clinical study. Key priorities include replication in additional preclinical systems (including patient-derived oligodendrocytes), identification of molecules with reliable CNS penetration, and incorporation of biomarkers sensitive to synuclein burden and glial dysfunction. Given MSA’s rapid progression, careful patient selection and well-powered trial design will be essential to detect potential disease-modifying effects.

### ALS.

ALS is a relentlessly progressive disorder characterized by upper and lower motor neuron loss, excitotoxicity, and glial activation. Given that GLP-1RAs enhance insulin signaling, reduce neuroinflammation, and support mitochondrial function, they represent a plausible therapeutic strategy for ALS.

Preclinical evidence is mixed. In toxin-based models, upregulation of GLP-1/IGF-1 signaling by 4-hydroxyisoleucine improved motor behavior, preserved myelin, and reduced apoptosis (166). In SOD (G93A) ALS mice, a clinically translatable dose of exendin-4 as well as an intracerebroventricular injection of GLP-1–secreting mesenchymal stromal cells improved motor function and preserved spinal motor neurons, accompanied by reduced glial activation (167). Conversely, liraglutide failed to alter survival or pathology in SOD1G93A and TDP-43Q331K mice, underscoring uncertainty over molecule choice, dosing, and CNS penetration (168).

Human data remain limited and contradictory. In 44 patients with ALS, endogenous GLP-1 (and insulin and amylin) levels correlated with slower disease progression and inversely with the proinflammatory chemokine MCP-1, suggesting a potential protective link (169). Retrospective studies are inconsistent. One small cohort reported shorter survival in ALS patients with T2DM prescribed GLP-1RAs (170), while a large real-world dataset suggested reduced ALS risk among GLP-1RA users (138). To date, no RCTs have been conducted.

In summary, preclinical and biomarker evidence suggest GLP-1RAs may be useful in ALS, but conflicting animal and observational data highlight major uncertainties. Important next steps include exploratory early-phase trials to establish CNS penetrance, target engagement (CSF GLP-1/IGF-1), and downstream biomarkers such as MCP-1 and NfL, before larger efficacy studies can be justified.

### HD.

HD is an autosomal dominant disorder caused by CAG repeat expansion in the huntingtin gene (*HTT*), leading to progressive motor and cognitive decline with no disease-modifying treatment. Mutant huntingtin (mHTT) induces metabolic and cellular stress through impaired insulin signaling, defective autophagy, mitochondrial dysfunction, oxidative stress, and synaptic loss (171), processes that overlap with pathways modulated by GLP-1RAs.

Preclinical studies do provide early support for a protective effect of GLP-1RAs. In cell models overexpressing mHTT, liraglutide restored insulin signaling, activated AMPK, upregulated antioxidant responses, and enhanced autophagy, reducing aggregate burden and improving viability (172). In a 3-nitropropionic acid toxin model, liraglutide improved motor performance, reduced oxidative stress, and activated PI3K/Akt/CREB/BDNF signaling (173). In N171-82Q transgenic mice, exenatide improved motor function, prolonged survival, normalized glucose metabolism, and reduced mHTT aggregation in brain and pancreatic tissue (58, 174). However, evidence remains sparse, with only a few transgenic studies and no human clinical data to date. Translation will require replication in additional genetic HD models and incorporation of patient-derived iPSC systems before early clinical trials can be justified.

### MS.

GLP-1RAs effects are relevant to MS, with potential to modulate inflammation, promote remyelination, and to stabilize the BBB. GLP-1Rs are expressed on oligodendrocytes and Schwann cells (175), lending further support for their potential use in demyelinating disorders (175).

Preclinical studies provide mixed evidence. Exenatide improved remyelination and functional recovery in peripheral nerve injury models (176–180); however, in a cuprizone mouse model, NLY01 failed to limit demyelination or promote remyelination (181). In contrast, in experimental autoimmune encephalomyelitis (EAE) models, exendin-4, liraglutide, and dulaglutide consistently attenuate inflammation and oxidative stress (81, 87, 182), reduce clinical severity, and improve motor outcomes (83), mediated by AMPK/SIRT1 activation and NLRP3 inhibition.

Human data remain scarce. A retrospective analysis indicated GLP-1RA use was associated with reduced MS incidence (138), and a small study of dulaglutide in 13 patients demonstrated preserved endothelial function over 12 months (183). However, no trials have evaluated GLP-1RAs against standard MS clinical endpoints such as relapse rate, MRI activity, or disability progression.

Overall, GLP-1RAs reliably reduce inflammation in MS models, but effects on remyelination are inconsistent and model-dependent. Future progress will require early-phase trials using traditional MS endpoints and biomarker-driven designs.

### Other neurological disorders: traumatic brain injury.

Across preclinical traumatic brain injury (TBI) models (concussive, blast, fluid percussion, and controlled cortical impact), GLP-1RAs consistently mitigate neuroinflammation, oxidative stress, and stabilize the BBB, resulting in functional improvements of cognitive and motor recovery (184–186). Some effects of TBI on gene expression are reversed by clinically translatable doses of exendin-4 (187, 188); exendin-4 is also associated with inhibition of MAPK signaling that leads to suppression of proinflammatory cytokines, restoration of mitochondrial function, and improved clearance of CSF toxic metabolites through enhanced glymphatic function (189, 190). Human data are very limited, but a recent study showed that individuals with TBI had elevated GLP-1 levels, which may be compensatory and suggest dysfunctional CNS GLP-1 signaling (191). Moreover, in a recent clinical evaluation of exendin-4 in individuals with idiopathic intracranial hypertension, an immediate (within 2.5 hours) decline in intracranial pressure was reported (192), in line with the expression of GLP-1Rs at the choroid plexus and involvement in CSF generation and regulation of intracranial pressure (193). Despite promising mechanistic and physiological actions, no interventional TBI trials have yet been conducted (194).

### Stroke.

GLP-1RAs show robust neuroprotection in ischemic stroke models, reducing infarct size and enhancing recovery when given both before and after insult (144, 195). Meta-analyses of cardiometabolic outcome trials of GLP-1RAs demonstrate approximately 15%–20% lower stroke incidence in people with diabetes and obesity (195–197), with recent data extending benefits to nondiabetic populations (198, 199). These effects reflect indirect cardiovascular risk reduction and direct neurovascular actions, including enhanced endothelial function and reduced neuroinflammation (196, 200). However, no trials have tested GLP-1RAs as stroke treatments or secondary prevention agents, so the relative contribution of CNS versus systemic mechanisms remains unclear. Nevertheless, this building evidence has triggered calls to explore GLP-1RAs as novel treatments to reduce stroke risk and improve neurological outcomes in at-risk populations (196, 198).

### Psychiatric disorders.

GLP-1RAs modulate oxidative stress, inflammation, and monoaminergic and mesolimbic circuits in preclinical models, resulting in functional improvements in depression and anxiety, and also reducing drug reward (51, 201, 202). Epidemiological and genetic data are supportive. Mendelian randomization studies suggest reduced risks of schizophrenia, bipolar disorder, PTSD, and eating disorders in individuals treated with GLP-1RAs (203). A recent meta-analysis of randomized trials in obesity and/or diabetes found no increased risk of psychiatric adverse events, with improvements in quality of life, restrained eating, and emotional regulation measures (204). Thus, GLP-1RAs represent promising candidates for psychiatric disorders — particularly addiction, depression, and cognitive impairment — but dedicated clinical trials are now required to determine efficacy and define optimal therapeutic windows.

## Challenges and opportunities in translating GLP-1RAs to neurodegeneration

### Beyond GLP-1: dual and triple agonists.

Since the development of single GLP-1RAs, several dual agonists (GLP-1/GIP; GLP-1/glucagon) and triple agonists (GLP-1/GIP/glucagon) are already approved or in advanced clinical development for diabetes and obesity, with translational studies now extending to neurodegeneration. These newer molecules consistently outperform single GLP-1RAs in regards to glycemic control and weight loss (205–207), although they exhibit similar gastrointestinal (GI) side effects. This superior systemic efficacy raises the possibility of stronger impact on neurodegenerative pathways that overlap with metabolic dysfunction, such as IR, mitochondrial stress, and inflammation.

Accordingly, preclinical AD and PD models suggest that dual and triple agonists may enhance neurotrophic and neuroprotective actions, while reducing Aβ and α-synuclein pathology, oxidative stress, and inflammation (49, 208, 209). Limited comparative data suggest dual and triple agonists outperform single agonists in key measures of neuroprotection and antiinflammatory response, positioning them as potentially more efficacious candidates for NDD therapy (209–213).

However, important translational challenges remain; brain penetrance varies widely between GLP-1RA–based drugs (214, 215), and evidence of the superiority of dual and triple agonists in modulating neurodegenerative pathways remains confined to cell and animal models. Whether these benefits translate into meaningful clinical efficacy in human NDDs remains unknown. A systematic translational roadmap will be required to realize their potential in NDDs. This would include comparative preclinical studies using standardized models of proteinopathy and metabolic dysfunction to define class-specific mechanisms, followed by early-phase human trials incorporating pharmacokinetic and biomarker endpoints of brain target engagement. Such work will clarify whether enhanced metabolic and antiinflammatory actions translate into measurable clinical benefit.

### CNS delivery and target engagement.

GLP-1RAs represent a pharmacologically diverse class, differing in receptor kinetics, molecular size, half-life, and capacity to cross the BBB, which likely contributes to variability in neuroprotective efficacy across compounds. Radiolabel studies suggest small peptides like exenatide and lixisenatide cross the BBB, while acylated or PEGylated analogues (liraglutide, semaglutide, NLY01) show minimal uptake. The clinical failure of NLY01 in PD, despite evidence of CNS penetration in nonhuman primates (158), underscores the uncertainty around how brain access relates to clinical efficacy.

Human trial data are inconsistent but suggest variability between drugs and formulations: exenatide (Bydureon) reached 1%–3% of peripheral levels in CSF (156), while another formulation of once-weekly exentide (Bcise) only achieved approximately 1% of peripheral levels detectable in CSF (61). While liraglutide does not seem to cross the BBB (216), improvements have been reported in AD and PD cohorts (97, 217).

A key unresolved issue is whether direct BBB penetration is required or whether therapeutic effects can arise from circumventricular activation, peripheral metabolic improvement, or systemic immunomodulation. Recent data also suggest that some antiinflammatory effects of GLP-1RAs may be centrally mediated even with minimal penetration (218). Adding further complexity, GLP-1RAs can stabilize the BBB (219, 220), raising the possibility that drugs with limited baseline uptake may improve their own CNS access over time, particularly in aging or NDD states where BBB dysfunction is common. It remains unclear whether GLP-1RAs primarily exploit disease-related BBB dysfunction or actively remodel the barrier to support neuroprotection.

### Safety, adherence, and patient stratification.

Although generally safe, GLP-1RAs cause consistent GI side effects that contribute to dose interruptions and discontinuations in obesity and diabetes trials. In older or frail populations, concerns include lean-mass loss, aspiration risk during procedures, sarcopenia, falls, and worsening dysphagia. Because weight loss is already common across NDDs, excessive or rapid GLP-1RA–induced weight loss may exacerbate frailty, potentially offsetting neurological benefits. Careful monitoring of nutrition and lean mass should therefore be integrated into NDD trials.

Beyond tolerability, adherence strongly influences outcomes. Real-world discontinuation rates for GLP-1RAs range from 17%–35% in the first year, varying by drug and country (221). These challenges are likely to be greater in older adults with cognitive or motor decline. Trials will therefore require structured support — slow titration, proactive side-effect management, nutritional and exercise guidance, and caregiver involvement — to prevent dilution of efficacy signals.

Even when treatment is continued, there remains great heterogeneity of response, exemplified by only approximately 50%–70% of patients achieving clinically meaningful weight loss outside of clinical trials (221, 222). Response rates vary by molecule — newer agents such as tirzepatide and semaglutide yield higher responder rates (~86%–91%) than older agents such as exenatide or liraglutide (~40%–65%) (223) — and vary by baseline BMI, sex, and glycemic status, with women and individuals with higher BMI responding better (222, 224). Genetic and pharmacogenomic studies implicate *GLP1R*, *ARRB1* (β-arrestin-1), and other loci in contributing to interindividual variability (225).

Despite years of clinical experience in diabetes and obesity, predicting responders to GLP-1RAs remains imprecise, closer to “tossing a coin” than true precision medicine (224, 226). Early exploratory work in NDDs suggests that individuals with evidence of brain or IR may benefit most (26), and post hoc analyses hint that higher BMI, younger age, T2DM comorbidity, and earlier disease stage might predict better response (227). Future NDD trials will likely need biological enrichment strategies (e.g., selecting BIR-positive or metabolically dysregulated individuals), combined with biomarker endpoints of central insulin signaling and stringent adherence frameworks, to reliably identify responders.

### Broader translational hurdles: outcome measures and trial design.

Traditional clinical endpoints often lack sensitivity to detect disease-modifying effects, particularly over short trial durations. Weight loss and GI side effects risk unblinding, potentially inflating placebo responses. Innovative designs incorporating active comparators, adaptive frameworks, and biomarker-driven endpoints (e.g., CSF/serum NfL, p-tau, microglial PET, synaptic markers) will be critical to generate interpretable efficacy signals.

### Timing of intervention.

In preclinical studies, GLP-1RAs are typically administered before or immediately after the onset of pathology, whereas clinical trials have enrolled mild-to-moderate symptomatic cohorts. Earlier intervention — at prodromal or preclinical stages — is likely to enhance efficacy. Future trials should include stage-stratified enrollment. Whether GLP-1RAs can modify disease once substantial neuronal loss has occurred remains uncertain, although benefits on later features (e.g., cognitive decline, gait or balance impairments) merit evaluation.

### Combination approaches and disease heterogeneity.

With anti-Aβ and anti-tau therapies entering AD practice, GLP-1RAs may be most effective in combination, targeting metabolic and inflammatory pathways that complement direct amyloid or tau lowering. Similarly, GLP-1RAs may offer greatest benefit in disorders with strong metabolic and proteinopathy links (AD, PD, MS), whereas effects in primarily RNA-driven diseases such as ALS or HD may be more limited. Disease-specific prioritization will be essential.

### Practical and economic considerations.

Injectable formulations may pose barriers for frail or cognitively impaired patients, and the demand for GLP-1RAs in obesity and diabetes creates challenges for scalable use in NDDs. Development of oral, depot, or transdermal delivery systems may improve adherence. Cost effectiveness and equitable access will need careful consideration if GLP-1RAs are to be deployed more broadly in NDDs. Emerging oral and long-acting formulations may overcome barriers to self-injection in frail or cognitively impaired populations.

## Conclusions

Across preclinical and translational studies, GLP-1RAs have emerged as promising candidates for NDD therapy, consistently modulating convergent mechanisms of pathology: IR, mitochondrial dysfunction, oxidative stress, proteostasis failure, synaptic loss, and neuroinflammation. Evidence for their potential application in AD and PD is strongest, with signals of target engagement in humans, while data in rarer disorders such as MSA, ALS, HD, and MS remain sparse and inconsistent. Observational studies suggest potential protective associations, but clinical trials to date show mixed efficacy, highlighting unresolved challenges around molecule selection, CNS penetration, tolerability, adherence, and patient stratification.

Progress will depend on biomarker-driven, biologically enriched trial designs capable of determining whether the pleiotropic benefits observed in experimental systems translate into meaningful disease modification.

A useful conceptual framework comes from exercise, one of the few interventions with proven disease-modifying influence in NDDs. Structured physical activity enhances vascular and metabolic health, increases neurotrophic signaling, strengthens synaptic plasticity, and bolsters mitochondrial resilience, with benefits documented across aging, AD, and PD populations (228–230). These pleiotropic actions mirror many attributed to GLP-1RAs — enhancing insulin signaling, reducing neuroinflammation, and stabilizing energy homeostasis. This parallel suggests a unifying therapeutic principle: GLP-1RAs may act as a “pharmacological analogue of exercise,” offering a complementary strategy where lifestyle interventions alone are insufficient or impractical. Framed in this way, the rationale for their continued development as disease-modifying therapies in NDDs is strong, but must now be matched by well-designed, biomarker-rich clinical trials.

## Funding support

This work is the result of NIH funding, in whole or in part, and is subject to the NIH Public Access Policy. Through acceptance of this federal funding, the NIH has been given a right to make the work publicly available in PubMed Central.

- Medical Research Council/National Institute for Health and Care Research (NIHR) Clinical Academic Research Partnership (to DA).
- Cure Parkinson’s Trust (to DA and TF).
- NIHR (to TF).
- MJFox Foundation (to TF and SG).
- John Black Charitable Foundation (to TF).
- Janet Owens Research fellowship (to TF).
- Edmond J Safra Foundation (to TF).
- Van Andel Institute (to TF).
- Defeat MSA (to TF).
- UCLH Biomedical Research Centre (TF and SG).
- MRC Senior Clinical Fellow, MR/T008199/1 (SG).

## Figures and Tables

**Table 1:**
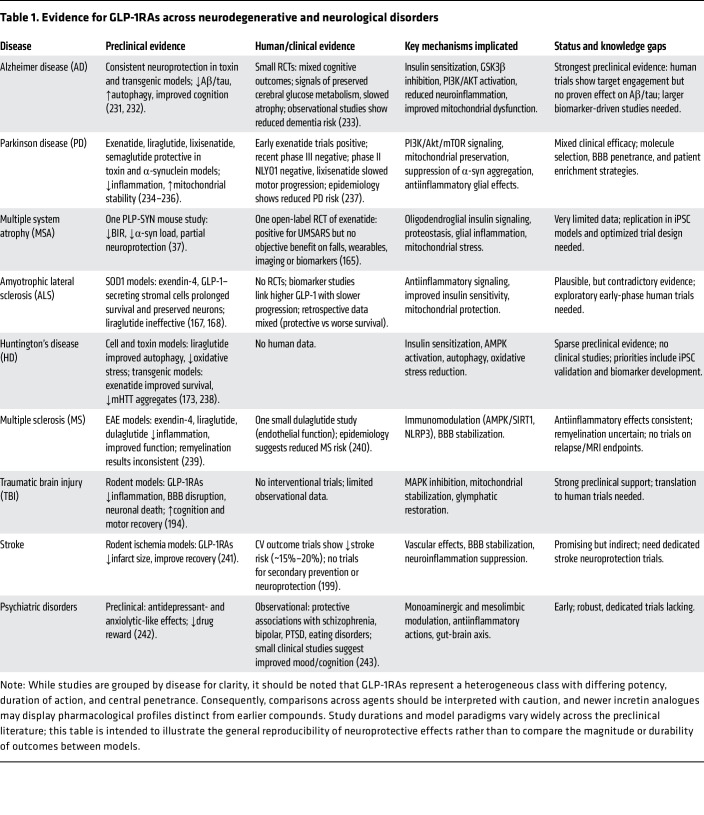
Evidence for GLP-1RAs across neurodegenerative and neurological disorders

## Version 1 02/16/2026

Electronic publication
